# Reactive oxygen species and tumor dissemination: Allies no longer

**DOI:** 10.1080/23723556.2015.1127313

**Published:** 2016-01-19

**Authors:** Cecilia Herraiz, Eva Crosas-Molist, Victoria Sanz-Moreno

**Affiliations:** Tumour Plasticity Laboratory, Randall Division of Cell and Molecular Biophysics, New Hunt's House, Guy's Campus, King's College London, London SE1 1UL, UK

**Keywords:** Amoeboid migration, antioxidants, cancer metastasis, DNA damage, PIG3, reactive oxygen species (ROS), RHO GTPases, ROCK, P53

## Abstract

For decades, reactive oxygen species (ROS) linked to oxidative stress have been suggested to promote carcinogenesis. However, we and others have demonstrated a protective role for ROS in metastatic dissemination. These recent studies partly explain the large failure observed in clinical trials using antioxidants for cancer prevention.

## Abbreviations

3D: 3-dimensional
ATM: ataxia-telangiectasia mutated
GSH: glutathione
MMP9: matrix metalloproteinase 9
NAC: N-acetyl-cysteine
PIG3: P53-inducible gene 3
ROCK: RHO-associated protein kinase
ROS: reactive oxygen species
TGF-β: transforming growth factor-beta

## 

Reactive oxygen species (ROS) or free radicals can damage DNA, potentially leading to genetic aberrations that can cause cancer. Antioxidants can counteract these free radicals and this should have a protective effect. Such a rationale has fuelled a profitable industry selling antioxidant supplements. A series of 3 recently published studies in melanoma, the most aggressive form of skin cancer, suggests otherwise.^1-3^ From different angles the 3 studies have reached the same conclusion: antioxidants increase metastatic success and metastasis is the cause of 90% of cancer deaths. Melanoma arises from melanocytes, which protect the skin from the mutagenic effects of ultraviolet radiation by producing melanin and causing a photoprotective tanning response but ROS are generated as by-products. As a result, melanocytes induce the DNA repair machinery and increase expression of antioxidant systems to decrease DNA damage and restore the normal redox state.^4^ Thus, melanocytes have a special and intricate relationship with ROS.

Cell migration is essential for melanoma cells to escape the primary tumor and metastasize. RHO family GTPases are key regulators of migration through their actions on the cytoskeleton. These enzymes are molecular switches that are active when bound to GTP. GTPase activating proteins, or GAPs, are therefore key inhibitors of RHO GTPase activity. Melanoma cells can migrate by adopting different strategies; among them, rounded-amoeboid migration relies on high levels of RHO and RHO-associated protein kinase (ROCK) leading to high actomyosin contractility.^5^ Furthermore, melanoma cells with high levels of RHO-ROCK-actomyosin have developed mechanisms to allow fast migration in physiological 3-dimensional (3D) environments and inhibit excessive RAC1-mediated cell adhesion.^5^ Therefore, rounded-amoeboid cells are very often found at the invasive front of melanomas.^5,6^ RAC1, on the other hand, is not only a key regulator of cell adhesion but is also crucial for controlling non-mitochondrial ROS via its actions of NADPH oxidases.^7^

Recent work from our laboratory was initiated to understand the consequences of reducing actomyosin contractility and decreasing cell migratory behavior on ROS metabolism. By decreasing the expression of ROCK and/or actomyosin we were able to increase RAC1 activity and, therefore, levels of intracellular ROS.^1^ Next, we showed that melanoma cells in the presence of hydrogen peroxide had lower actomyosin levels and higher RAC1 activity, which resulted in reduced invasiveness through a 3D matrix. Importantly, using a panel of antioxidants we could increase actomyosin contractility in melanoma cells, and this resulted in very efficient 3D invasion. We could also measure oxidative stress-induced DNA damage resulting from the increase in ROS induced by lowering contractility, which resulted in activation of ataxia-telangiectasia mutated (ATM) and TP53, best known as p53. Downstream of p53, a transcriptional profile involving genes with roles in ROS metabolism and/or DNA damage was induced. This set of genes included an interesting p53 transcriptional target, P53-inducible gene 3 (*PIG3*). PIG3 has been implicated in DNA damage responses when localized in the nucleus but also harbors ROS producing activity as it is an oxido-reductase.^8^ This catalytic activity accounts for some of its functions as a pro-apoptotic gene. Interestingly, we found that the ability of PIG3 to produce ROS suppressed RHO GTP via activation of the RHO GAP, ARHGAP5. These results support the notion that antioxidants will favor actomyosin contractility and invasive behavior through inactivation of ARHGAP5, and therefore promotion of RHO activity. Using *in vivo* intravital imaging of melanoma tumors grown in mice we found that high levels of PIG3 in melanoma cells resulted in significantly less efficient dissemination. Furthermore, publicly available data extracted from The Cancer Genome Atlas showed that PIG3 levels were decreased, whereas ROCK1 and ROCK2 levels were increased, in human metastatic melanoma lesions compared to the primary tumors. Consistent with these results, we analyzed PIG3 protein levels using tissue microarrays and showed that 80% of melanoma samples had low or absent PIG3, and that cells in these samples had cytoskeletal features indicative of high contractility levels. Interestingly, a recent article by Le Gal et al.^2^ shows how administration of the general ROS scavenger N-acetyl-cysteine (NAC) in a melanoma mouse model increased lymph node metastasis. Both NAC and Trolox increased the migratory and invasive properties of human melanoma cells through increased glutathione (GSH) synthesis and increased activation of RHOA.^2^ This work completely supports our data. Importantly, in our study^1^ we found that oxidative stress-induced DNA damage responses can suppress the actomyosin machinery to impair migration of DNA-damaged cells. This could imply that melanoma cells that are repairing DNA may halt migration until the DNA damage has been resolved. Our findings also suggest that use of ROCK inhibitors downstream of RHO to reactivate p53 and PIG3 could be a useful therapeutic strategy. Further work is warranted to understand the consequences of long-term inhibition of actomyosin in melanoma.

Along these lines, a third study carried out by Piskounova et al.^3^ shows how melanoma metastasis is promoted by antioxidants *in vivo* after daily administration of NAC. Circulating melanoma cells are subjected to oxidative stress that limits distant metastasis, and only those cells able to reprogram their metabolism to increase GSH regeneration and withstand oxidative stress can survive and metastasize. Such GSH regeneration is possible thanks to NADPH generated from the folate pathway.

**Figure 1.**
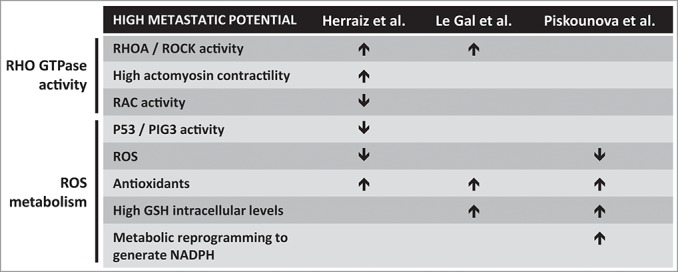
Impact of redox metabolism on the metastatic potential of cancer cells. Three recent reports studied regulation of the metastatic potential of melanoma cells in relation to redox cellular status. Common and specific observations from the three studies are summarized. GSH, glutathione; PIG3, P53-inducible gene 3; ROCK, RHO-associated protein kinase; ROS, reactive oxygen species.

Therefore, the 3 studies by Herraiz et al.,^1^ Le Gal et al.,^2^ and Piskounova et al.^3^ (Fig. 1), put together different pieces of a puzzle defining a scenario where ROS/antioxidants, through their effects on RHO GTPases and metabolic reprogramming, regulate metastatic potential and provide an explanation for the overall failure of clinical trials using antioxidants. Importantly, some cancer treatments, such as chemotherapy, rely on the actions of ROS on DNA to cause irreparable damage and lead to death of the cancer cells. Antioxidants could also interfere with such treatments.

In addition to these reports showing the relevance of antioxidants for cancer dissemination, a recent study in breast cancer by Harris et al.^9^ showed how antioxidant pathways are implicated not only in metastasis but also in cancer initiation and progression. Inhibition of GSH synthesis was sufficient to impair cancer initiation. However, once transformation had occurred alternative pathways were able to compensate for the lack of GSH, making it necessary to block different antioxidants.^9^ Inhibition of GSH synthesis induced accumulation of glutamate, which was exported in exchange for cysteine via an amino acid transporter system composed of the XCT subunit and CD44, thus potentiating antioxidant synthesis when GSH was depleted.^9^ Interestingly, our group described how CD44 promotes rounded-amoeboid migration in melanoma.^6^ CD44-binding matrix metalloproteinase 9 (MMP9) favors melanoma amoeboid migration and actomyosin contractility.^6^ Transforming growth factor-β (TGF-β) also supports actomyosin-driven amoeboid migration.^10^ Since MMP9 can be activated by TGF-β, future studies will determine how all these molecules are related to CD44, antioxidant potential, and migratory behavior.

Understanding how different cancers respond to redox imbalances will be of the utmost importance. We need to understand how we can harness the power of free radicals as signaling intermediates, so that we can precisely manipulate the different sources of ROS. This would allow us to prevent the putative damage caused by oxidative stress without allowing adaptation of the tumor cells to such stress conditions. We are starting to unveil the complexities behind redox biology in cancer, but one question remains: Are antioxidants doing more harm than good in cancer prevention and as cancer therapies?

## References

[1] Herraiz C, Calvo F, Pandya P, Cantelli G, Rodriguez-Hernandez I, Orgaz JL, Kang N, Chu T, Sahai E, Sanz-Moreno V Reactivation of p53 by a Cytoskeletal Sensor to Control the Balance Between DNA Damage and Tumor Dissemination. J Natl Cancer Inst 2016; 108:djv289. 26464464 10.1093/jnci/djv289 PMC4712681

[2] Le Gal K, Ibrahim MX, Wiel C, Sayin VI, Akula MK, Karlsson C, Dalin MG, Akyurek LM, Lindahl P, Nilsson J, et al. Antioxidants can increase melanoma metastasis in mice. Sci Transl Med 2015; 7:308re8; PMID:26446958; http://dx.doi.org/10.1126/scitranslmed.aad3740 26446958

[3] Piskounova E, Agathocleous M, Murphy MM, Hu Z, Huddlestun SE, Zhao Z, Leitch AM, Johnson TM, DeBerardinis RJ, Morrison SJ. Oxidative stress inhibits distant metastasis by human melanoma cells. Nature 2015; 527:186-91; PMID:26466563; http://dx.doi.org/10.1038/nature15726 26466563 PMC4644103

[4] Denat L, Kadekaro AL, Marrot L, Leachman SA, Abdel-Malek ZA. Melanocytes as instigators and victims of oxidative stress. J Invest Dermatol 2014; 134:1512-8; PMID:24573173; http://dx.doi.org/10.1038/jid.2014.65 24573173 PMC4418514

[5] Sanz-Moreno V, Gadea G, Ahn J, Paterson H, Marra P, Pinner S, Sahai E, Marshall CJ. Rac activation and inactivation control plasticity of tumor cell movement. Cell 2008; 135:510-23; PMID:18984162; http://dx.doi.org/10.1016/j.cell.2008.09.043 18984162

[6] Orgaz JL, Pandya P, Dalmeida R, Karagiannis P, Sanchez-Laorden B, Viros A, Albrengues J, Nestle FO, Ridley AJ, Gaggioli C, et al. Diverse matrix metalloproteinase functions regulate cancer amoeboid migration. Nat Commun 2014; 5:4255; PMID:24963846; http://dx.doi.org/10.1038/ncomms5255 24963846 PMC4118761

[7] Hobbs GA, Zhou B, Cox AD, Campbell SL. Rho GTPases, oxidation, and cell redox control. Small GTPases 2014; 5:e28579; PMID:24809833; http://dx.doi.org/10.4161/sgtp.28579 24809833 PMC4114927

[8] Porte S, Valencia E, Yakovtseva EA, Borras E, Shafqat N, Debreczeny JE, Pike AC, Oppermann U, Farres J, Fita I, et al. Three-dimensional structure and enzymatic function of proapoptotic human p53-inducible quinone oxidoreductase PIG3. J Biol Chem 2009; 284:17194-205; PMID:19349281; http://dx.doi.org/10.1074/jbc.M109.001800 19349281 PMC2719357

[9] Harris IS, Treloar AE, Inoue S, Sasaki M, Gorrini C, Lee KC, Yung KY, Brenner D, Knobbe-Thomsen CB, Cox MA, et al. Glutathione and thioredoxin antioxidant pathways synergize to drive cancer initiation and progression. Cancer Cell 2015; 27:211-22; PMID:25620030; http://dx.doi.org/10.1016/j.ccell.2014.11.019 25620030

[10] Cantelli G, Orgaz JL, Rodriguez-Hernandez I, Karagiannis P, Maiques O, Matias-Guiu X, Nestle FO, Marti RM, Karagiannis SN, Sanz-Moreno V. TGF-β-Induced Transcription Sustains Amoeboid Melanoma Migration and Dissemination. Curr Biol 2015; 25(22):2899-914; PMID:26526369 26526369 10.1016/j.cub.2015.09.054 PMC4651903

